# FOXO3a-ROS pathway is involved in androgen-induced proliferation of prostate cancer cell

**DOI:** 10.1186/s12894-022-01020-9

**Published:** 2022-04-29

**Authors:** Yan Tao, Shanhui Liu, Jianzhong Lu, Shengjun Fu, Lanlan Li, Jing Zhang, Zhiping Wang, Mei Hong

**Affiliations:** 1 Key Laboratory of Urological Disease in Gansu Province, Lanzhou University Second Hospital, No. 82 Cuiyingmen, Lanzhou, 730030 Gansu China; 2 Institute of Gansu Nephron-Urological Clinical Center, Lanzhou University Second Hospital, Lanzhou, Gansu China; 3 State Key Laboratory of Chemical Oncogenomics, School of Chemical Biology and Biotechnology, Peking University Shenzhen Graduate School, Nanshan District, Shenzhen, 518055 Guangdong China; 4 Key Laboratory of Chemical Genomics, School of Chemical Biology and Biotechnology, Peking University Shenzhen Graduate School, Shenzhen, Guangdong China; 5 Drug Discovery Center, School of Chemical Biology and Biotechnology, Peking University Shenzhen Graduate School, Shenzhen, Guangdong China

**Keywords:** Prostate cancer, FOXO3a, ROS, Catalase

## Abstract

**Background:**

Although FOXO3a can inhibit the cell proliferation of prostate cancer, its relationship with reactive oxygen species (ROS) in prostate cancer (PCa) has not been reported.

**Methods:**

We analyzed the correlation between the expression of *FOXO3a* and the antioxidant enzyme *catalase* in prostate cancer with the TCGA and GEPIA databases. We also constructed a PPI network of FOXO3a via the STRING database. The mRNA and protein expression of FOXO3a and catalase were detected by qRT-PCR or western blotting in LNCaP and 22RV1 cells treated with DHT, R1881, or Enzalutamide. The effects of FOXO3a on catalase expression were tested by over-expressing or knocking down FOXO3a in LNCaP cells. Furthermore, the catalase activity and ROS level were detected in LNCaP cells treated with DHT. Cell proliferation and ROS were also analyzed in LNCaP which was treated with antioxidant.

**Results:**

Results showed that the *catalase* expression was down-regulated in prostate cancer. A positive correlation between *FOXO3a* and *catalase* existed. DHT treatment could significantly reduce FOXO3a and catalase expression at mRNA and protein level in LNCaP cells. Catalase expression partly depended on FOXO3a as over-expression and knockdown of *FOXO3a* could result in the expresssion change of catalase. DHT treatment was found to inhibit catalase activity and increase ROS level in prostate cancer cell. Our study also demonstrated that antioxidant treatment reduced DHT-induced proliferation and ROS production in prostate cancer cell.

**Conclusions:**

We discovered a novel mechanism by which DHT promotes prostate cancer cell proliferation via suppressing catalase activity and activating ROS signaling via a FOXO3a dependent manner.

**Supplementary Information:**

The online version contains supplementary material available at 10.1186/s12894-022-01020-9.

## Background

Prostate cancer (PCa) is one of the most common malignancies of the male genitourinary system [1]. Similarly, its incidence has been steadily increasing in China [2]. Patients with prostate cancer could benefit from androgen deprivation therapy. However, most patients inevitably progress to resistance against these treatment and developt to castration-resistant prostate cancer (CRPC) [3]. Therefore, understanding the molecular mechanisms leading to CRPC and identifying related molecular pathways are important for CRPC therapy.

Forkhead box O transcription factors (FOXO) regulate multiple cellular processes, including cell cycle arrest, cell death, DNA damage repair, stress resistance and metabolism [4]. Emerging evidence revealed FOXOs functions are arrested in prostate cancer. FOXOs may be a tumor suppressor in prostate cancer [5]. In PCa cells, numerous therapies could trigger cell growth arrest by suppressing the activity of FOXO3a (FOXO3) [6–8]. Studies have also shown that oxidative stress is associated with the progression of prostate cancer in an androgen-dependent manner or an androgen-independent manner [9]. Oxidative stress regulates multiple cellular processes, such as cell growth and apoptosis. FOXO3a was involved in antioxidative protection in cells via regulating the cell detoxification to reactive oxygen species(ROS) [10, 11]. However, whether the FOXO3a-ROS pathway plays an important role in the generation of oxidative stress and the progression in prostate cancer remains unknown. In the current study, we found that FOXO3a-ROS pathway was involved in the DHT inducing cell proliferation. DHT treatment could arrest the expression of FOXO3a and inhibit the activivty of catalase, which in turn increased ROS levels of PCa cell.

## Methods

### Gene expression analysis

To analyze the gene expression of *FOXO3a* (*FOXO3*) and *catalase* (*CAT*) in prostate cancer and in adjacent tissue, the relevant expression values of the genes were downloaded from The Cancer Genome Atlas (TCGA, https://cancergenome.nih.gov/). 52 normal prostate tissue sample and 499 prostate tumor sample were contained in the database. Wilcoxon rank sum test was used to evaluate the expression of *FOXO3* and *CAT* mRNA between prostate cancer tissues and unpaired tissues. Wilcoxon signed rank test was applied to evaluate the expression of *FOXO3a* and *CAT* mRNA between prostate cancer tissues and paired tissues. Immune infltrates score was calculate with GSVA package. The relationship beween immune inflatration and *FOXO3a* and *CAT* expression were estimated with Spearman correlation test.

### Correlation analysis between *FOXO3a* and *CAT*

Herein, we used GEPIA (http://gepia.cancer-pku.cn/index.html) [12] to analyze the correlation between *FOXO3a* expression and *catalase* expression in PCa. The correlation module generated the expression scatter plots between a pair of user-defined genes in PCa, together with the estimated statistical significance and the spearman’s correlation. Besides, we construct the FOXO3a protein–protein interaction (PPI) network analysis with STRING (https://string-db.org/).

### Cell culture and FOXO3a knockdown or over-expression

Human prostate cancer cell LNCaP and 22RV1 were obtained from the Chinese Academy of Sciences Cell Bank (Shanghai, China). Cells were cultured in the RPMI- 1640 medium (HyClone) supplemented with 10% FBS (HyClone), 100 U/ml penicillin and 100 μg/ml streptomycin. LNCaP cell was also cultured in the RPMI-1640 with 10% charcoal-stripped fetal bovine serum (Biological Industries). All cell lines were cultured in an incubator with 5% CO_2_ at 37 °C. For silencing *FOXO3a* expression, cells were transiently transfected with 100 pM of *FOXO3a*-specific siRNA and negative control siRNA (NC) which were designed and synthesized by GenePharma (Shanghai, China). Sequences of *FOXO3a* siRNAs were as follows: siRNA1 5′-GCUGUCUCCAUGGACAAUATT-3′; siRNA2 5′- GCACAGAGUUGGAUGAAGUTT-3′; negative control siRNA: sense 5′-UUCUCCGAACGUGUCACGUTT-3′, antisense 5′-ACGUGACACGUUCGGAGAATT-3. Cells were using Lipofectamine 2000 transfection reagent (Invitrogen 11668-019) following the manufacturer's protocol. To overexpress *FOXO3a* in prostate cancer cell line, we purchased FOXO3a vector and control vectors from GeneChem (Shanghai, China). The recombinant plasmid vector transfection was performed according to the protocol provided by the company. After incubating transfected cells for 6 h, the medium was replaced with fresh RPMI1640 medium containing 10% FBS. 24 h later, the transfected cells were stimulated with DHT or enzalutamide for 48 h. Cells were harvested for quantitative real-time-PCR (qRT-PCR) and western blotting to assess the gene expression of FOXO3a and catalase.

### Cell proliferation assays

5 × 10^3^ cells/well in 100 μl RPMI1640 medium supplemented with 10% FBS were seeded into 96-well plates and incubated at 37 °C with 5% CO_2_ for 24 h. Then cells were stimulatedwith DHT (1, 10, 50 nM), R1881 (0.02, 0.1, 1 nM) (Sigma-Aldrich Chemicals) and Enzalutamide (40 μM, 60 μM) (Top Science, Shanghai, China), or DHT (10 nM) and the ROS scavenger Tiron (1 mM) (4,5-dihydroxy-1,3-benzenedisulfonic acid, disodium salt monohydrate; Sigma-Aldrich Chemicals) for another 48 h. The cell proliferation was measured by adding 10 μl CCK8 (DojinDo, Japan) into each well and incubating at 37 °C for 4 h. Absorbance at 450 nm wavelength was measured by multifunctional chemiluminescence detector(Berto,Germany).

### RNA isolation and qRT-PCR

Total RNA was extracted with TRIzol reagent (Takara Biotechnology Co., Dalian, China), and reverse transcribed using the PrimeScript RT reagent kit (Takara, Dalian, China). The target genes were quantified by quantitative real-time PCR using the SYBR Premix Ex Taqkit (Takara, Dalian, China) in a real-time thermal cycler ( Bio-Rad Laboratories, Inc., USA). All mRNA expression levels were normalized to the β-actin. The primer sequences used were: FOXO3a (forward: 5′- CTACGAGTGGATGGTGCGTT-3′; Reverse: 5′- TCTTGCCAGTTCCCTCATTC-3′); Catalase (forward: 5′- AGATGCAGCACTGGAAGGA-3′; Reverse: 5′- CACGGGGCCCTACTGTAATA-3′); PSA (forward: 5′- CTGTCAGAGCCTGCCGAG-3′; Reverse: 5′- CTGGTTCAATGCTGCCCC-3′); TMPRSS2 (forward: 5′- TATGAAACTGAACACAAGTG-3′; Reverse: 5′- GCTATACAGCGTAAAGAAAC-3′); β-actin (forward: 5′- GCACAGAGCCTCGCCTT-3′; Reverse: 5′- GTTGTCGACGACGAGCG-3′).

### Immunoblotting

Prostate cancer cell lines stimulated in vitro as indicated above were lysed in the cell lysis buffer (Beyotime Biotechnology, China). The lysated were quantified with the bicinchoninic acid (BCA) kit (Beyotime Biotechnology, China). Then, the supernatants were subjected to 10% SDS-PAGE and then transferred to PVDF membranes (0.22 μm, Millipore) for detection of FOXO3a, catalase and β-actin. The blots were cut prior to hybridization with antibodies during blotting. Rabbit anti-FOXO3a (catalog no. 12829) was purchased from Cell Signaling Technology. Rabbit anti-catalase (catalog no. sc-50508) was from Santa Cruz Biotechnology. β-actin (TA-09) was from ZSGB-BIO (Beijing, China) and dye-labeled secondary antibody (IRDye800 or IRDye700) was from LI-COR. Band intensity was quantified using the Image Studio Software.

### Catalase activity detection

Catalase activity was detected with a catalase analysis kit (Beyotime Biotechnology, China) according to the manufacturer's instructions. Briefly, The cell lysates were treated with excess hydrogen peroxide for an indicated time, and then the remaining hydrogen peroxide (not decomposed by catalase) was coupled with a substrate that on treatment with peroxidase produced N-4-antipyryl-3-chloro-5-sulfonate-p-benzoquinonemonoimine, which has an absorption maximum at 520 nm and was quantified spectrophotometrically.

### ROS detection

5 × 10^3^ cells in 100 μl medium were seeded into 96-well plates per well and incubated at 37 °C with 5% CO_2_ for 24 h. Then, cells were cultured with DHT (10 nM) or/and Tiron (1 mM) for another 48 h. After washed two times with RPMI1640 medium, cells were incubated with DCFH-DA (Sigma-Aldrich Chemicals) for 30 min at 37 °C. Finally, fluorescence intensity was measured at 485 nm for excitation and 535 nm for emission.

### Statistical analysis

Data are presented as mean ± SEM. Statistical analysis was performed using two-tailed Student’s t-tests. Values of *p* < 0.05 were considered significant. Data analyses were performed using GraphPad Prism7.0.

## Results

### The mRNA expression levels of *FOXO3a* and *catalase* in prostate cancer

**Fig. 1:**
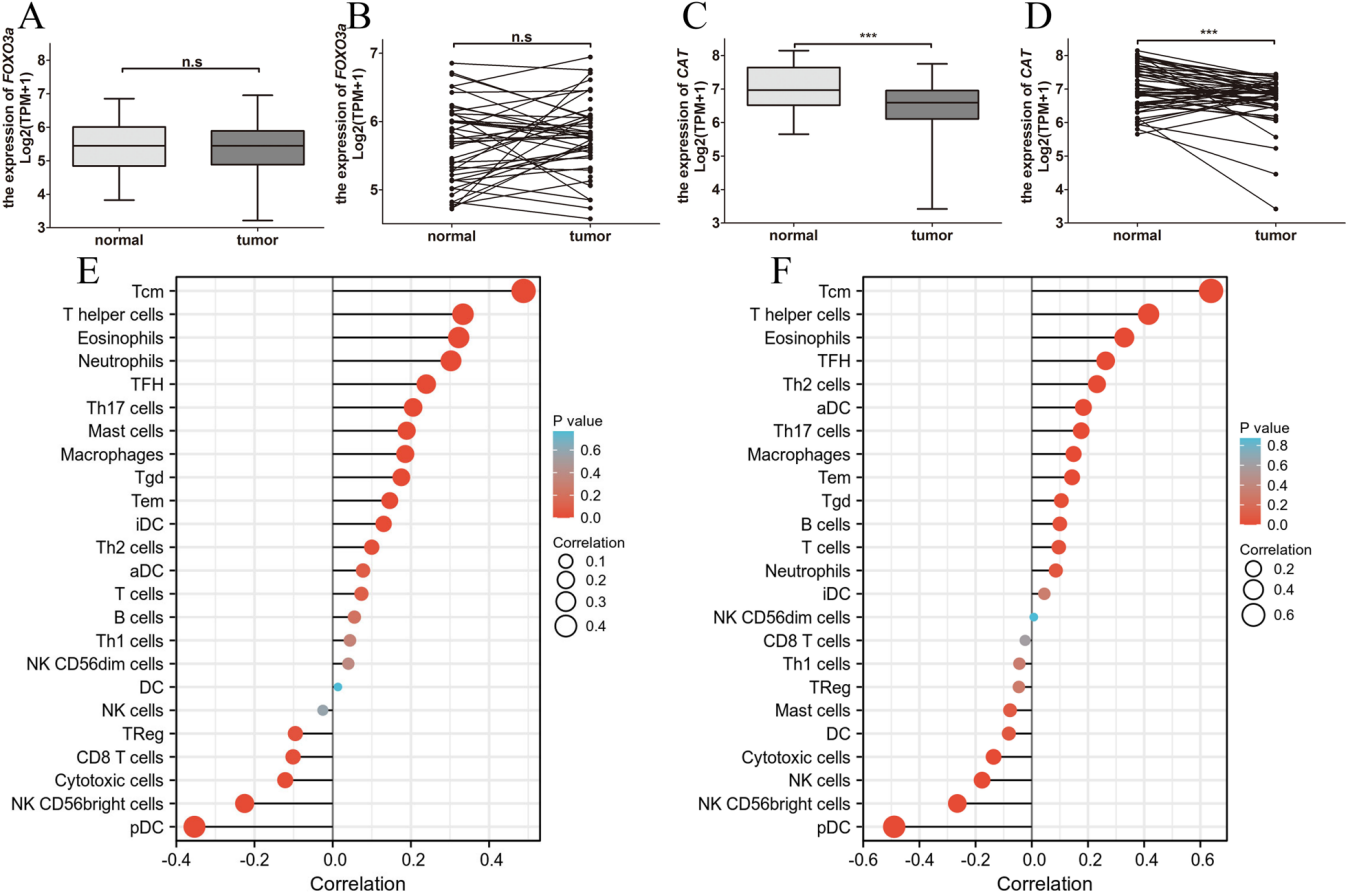
Relative epression level of *FOXO3a* and *catatlase* and the correlation to immune infiltrates. **A**, **C** Boxplot of *FOXO3a* and *catalase* mRNA expression between the prostate cancer and normal tissues in TCGA dataset (Normal = 52, Tumor = 499); **B**, **D** Pairwise boxplot of *FOXO3a* and *catalase* mRNA expression between the prostate cancer and normal tissues in TCGA datase (Normal = 52, Tumor = 52). Correlation between the abundance of immune infiltrates and *FOXO3a* expression (**E**) and *catalase* (**F**) in prostate cancer. (***p < 0.001)

To determine the differences of *FOXO3a* and *catalase* expression in prostate cancer and normal tissues, the *FOXO3a* and *catalase* mRNA levels were analyzed with the RNA-seq data from TCGA database. Result showed that the *catalase* expression was significantly reduced in prostate cancer compared with normal tissues (Fig. 1C, D). Although the *FOXO3a* expression was lower in prostate cancer, there was no significant difference between prostate cancer and normal tissues (Fig. 1A, B). We also assessed the correlation between immune infiltrates and *FOXO3a* and *catalase* expression in prostate cancer. Results indicated that *FOXO3a* and *catalase* expression is correlated with Tcm, T helper cells, etc. (Fig. 1E, F).

### The correlation between *FOXO3a* and *catalase*

**Fig. 2:**
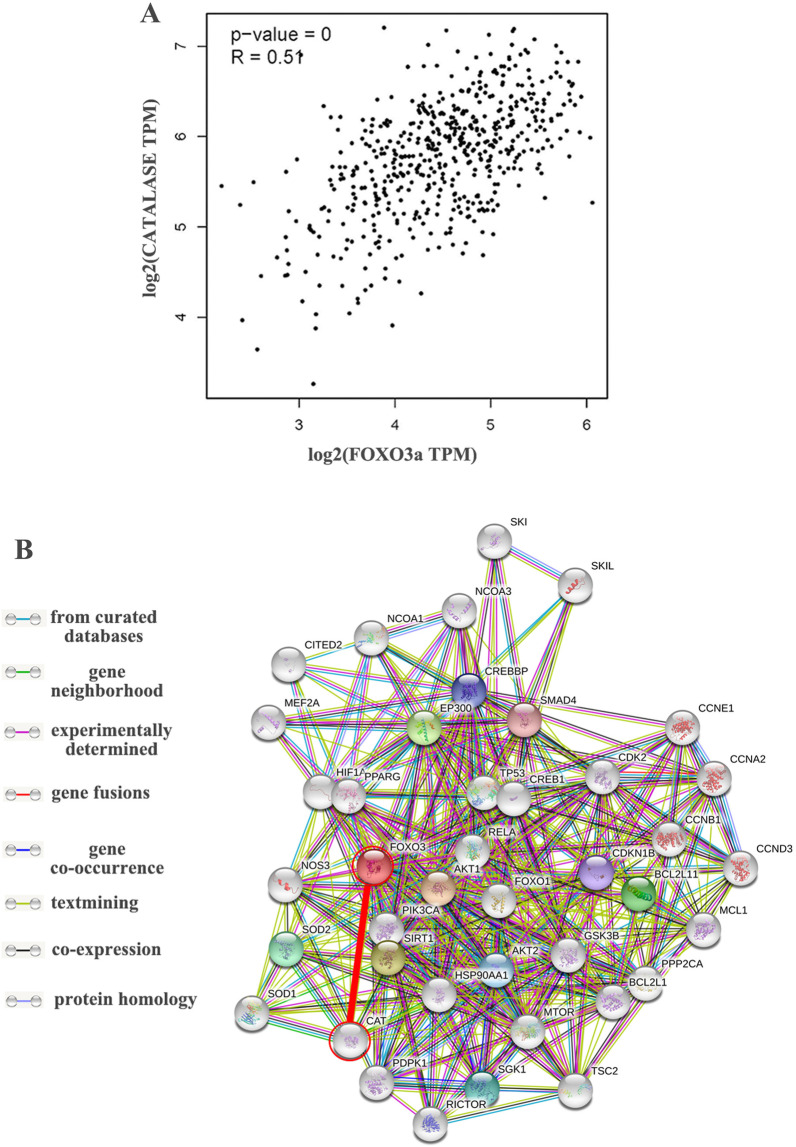
The correlation between FOXO3a and catalase at the gene and protein levels. **A** Scatterplots of correlations between *FOXO3a* expression and *catalase* in PCa by GEPIA database. **B** The PPI network, which contained 41 nodes, was constructed using the STRING database.The target protein (FOXO3a and catalase) has been marked in red

To further explore the correlation between *FOXO3a* and *catalase* in PCa, we evaluated the correlation in the GEPIA database [13, 14]. As shown in Fig. 2A, the expression of *FOXO3a* was significantly positively correlated with *catalase* in PCa (*p* < 0.001). To studying the function of FOXO3a, a PPI network was constructed using the STRING database. A total of 40 FOXO3a-interacting proteins were included in the PPI network complex and the resulting PPI network contained 41 nodes (Fig. 2B). As shown in Fig. 2B, FOXO3a has an interaction with catalase (CAT).

### DHT treatment down-regulated the expression of FOXO3a and catalase in the LNCaP cells

**Fig. 3:**
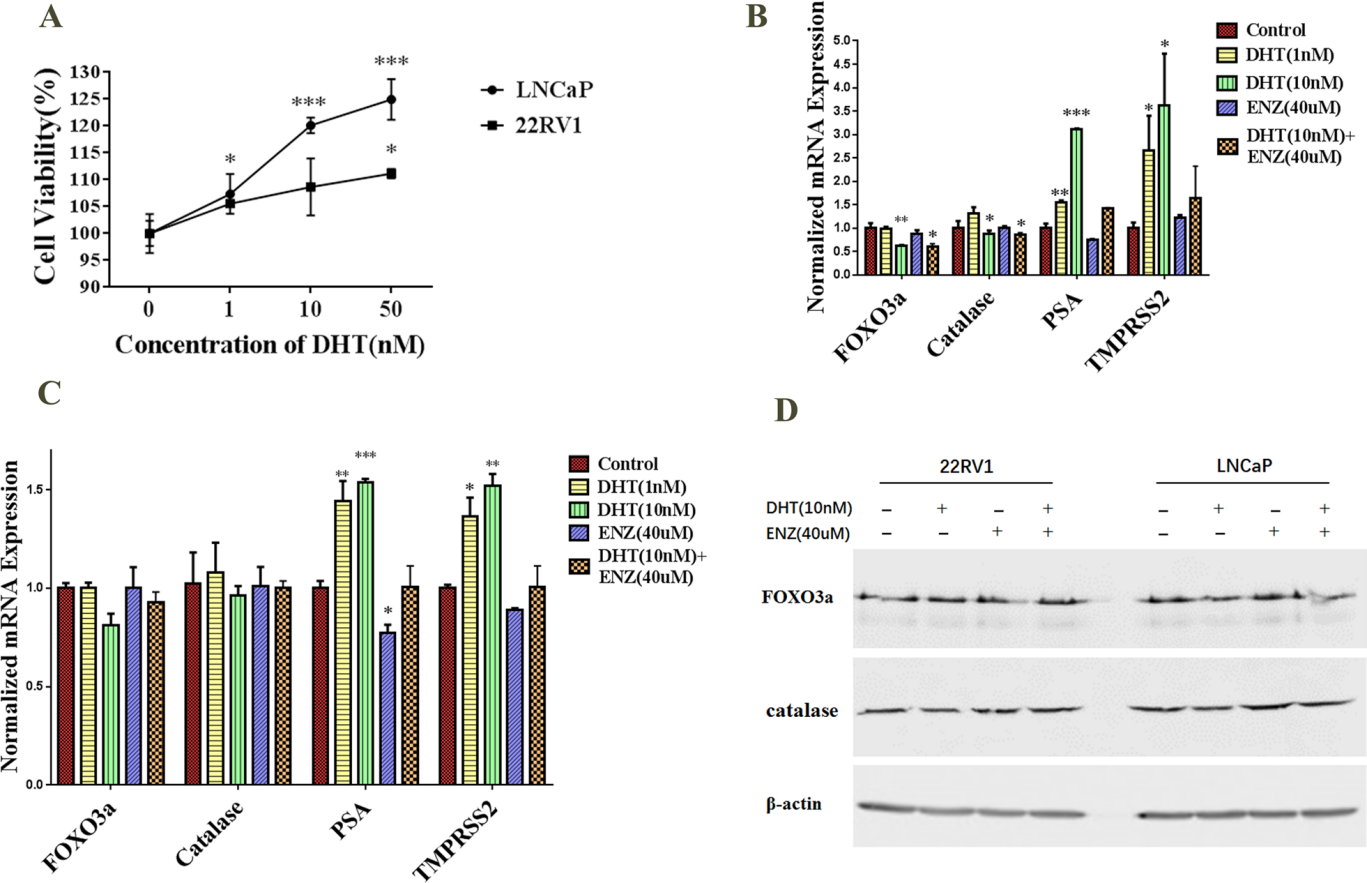
Activating androgen receptor decrease the expression of FOXO3a and catalase in LNCaP cells. **A** DHT treatment promoted proliferation in LNCaP and 22RV1 cells by CCK8 assay. **B** FOXO3a and catalase expression decreased in LNCaP cells which was treated with DHT. **C** FOXO3a and catalase expression were not change in 22RV1 cells which was treated with DHT. **D** DHT treatment down-regulated the expression of FOXO3a and catalase at protein level in LNCaP cells. (*p < 0.05, **p < 0.01, ***p < 0.001)

Previous studies have demonstrated that FOXO3a is down-regulated in prostate cancer [6]. To study whether FOXO3a and catalase are involved in the DHT and R1881 inducing prostate cancer proliferation, LNCaP and 22RV1 cells were treated with different concentrations of DHT or R1881 and Enzalutamide for 48 h, and cell viability was evaluated by the CCK8 assay. As shown in Fig. 3A and Additional file 1: Fig. S1, a significant increase in the proliferation effect was detected in a dose-dependent manner after treatment with DHT or R1881. Enzalutamide (40 μM or 60 μM) could slightly reduce the proliferation of LNCaP and 22RV1 cells. DHT and R1881 could reverse the proliferation effect which induced by Enzalutamide (Additional file 1: Fig. S1 and Additional file 2: Fig. S2). Results showed that the mRNA and protein levels of FOXO3a and catalase were indeed significantly down-regulated after the treatment with DHT (10 nM) in the LNCaP cells (Fig. 3B, D). However, FOXO3a and catalase expression change were not observed in 22RV1 cells which was treated with DHT (Fig. 3C, D). As a positive control, PSA and TMPRSS2 were found significantly up-regulated after treatment with DHT in LNCaP and 22RV1 cells (Fig. 3B, C). Enzalutamide could decreased the expression of AR-activated genes (Fig. 3B, C).

### DHT inducing catalase expression change depends on FOXO3a

**Fig. 4:**
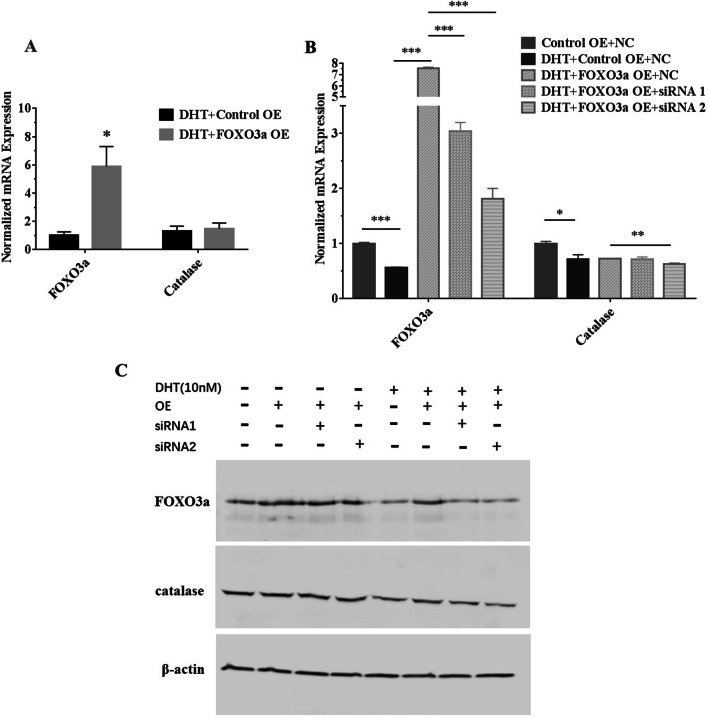
The effect of over-expression of FOXO3a and knockdown of FOXO3a on the catalase expression in LNCaP cells. **A** Over-expression of FOXO3a increased the expression of FOXO3a and catalase after DHT treatment in LNCaP cells. **B**, **C** FOXO3a siRNAs transfection can decrease the elevated expression of FOXO3a and catalase at mRNA and protein level after DHT treatment and over-expression of FOXO3a. Control OE, over-expressing control vector; FOXO3a OE, FOXO3a over-expressingvector; NC, negative control siRNA; siRNA1, first FOXO3a-specific siRNA; siRNA2, second FOXO3a-specific siRNA. (*p < 0.05, **p < 0.01, ***p < 0.001)

To explore whether FOXO3a affected the expression of catalase in LNCaP cells, the recombinant plasmid vectors containing FOXO3a fragment were transfected into the LNCaP cells. We found that the expression of FOXO3a was increased significantly (*p* < 0.05) (Fig. 4A, C). Although DHT treatment decreased catalase expression in LNCaP cells, over-expression of FOXO3a can restore the expression of catalase (Fig. 4A, C).

To further investigate whether FOXO3a knockdown can suppress the expression of catalase, two FOXO3a siRNAs were transfected into LNCaP cells. We found that both of FOXO3a siRNAs reduced the elevated expression of FOXO3a by over-expression vector (Fig. 4B, C). In addition, FOXO3a siRNAs also decreased the elevated expression of catalase (Fig. 4B, C). These results indicate that FOXO3a plays a vital role in the DHT regulating catalase expression.

### DHT treatment suppressed the catalase activity in LNCaP cells

**Fig. 5:**
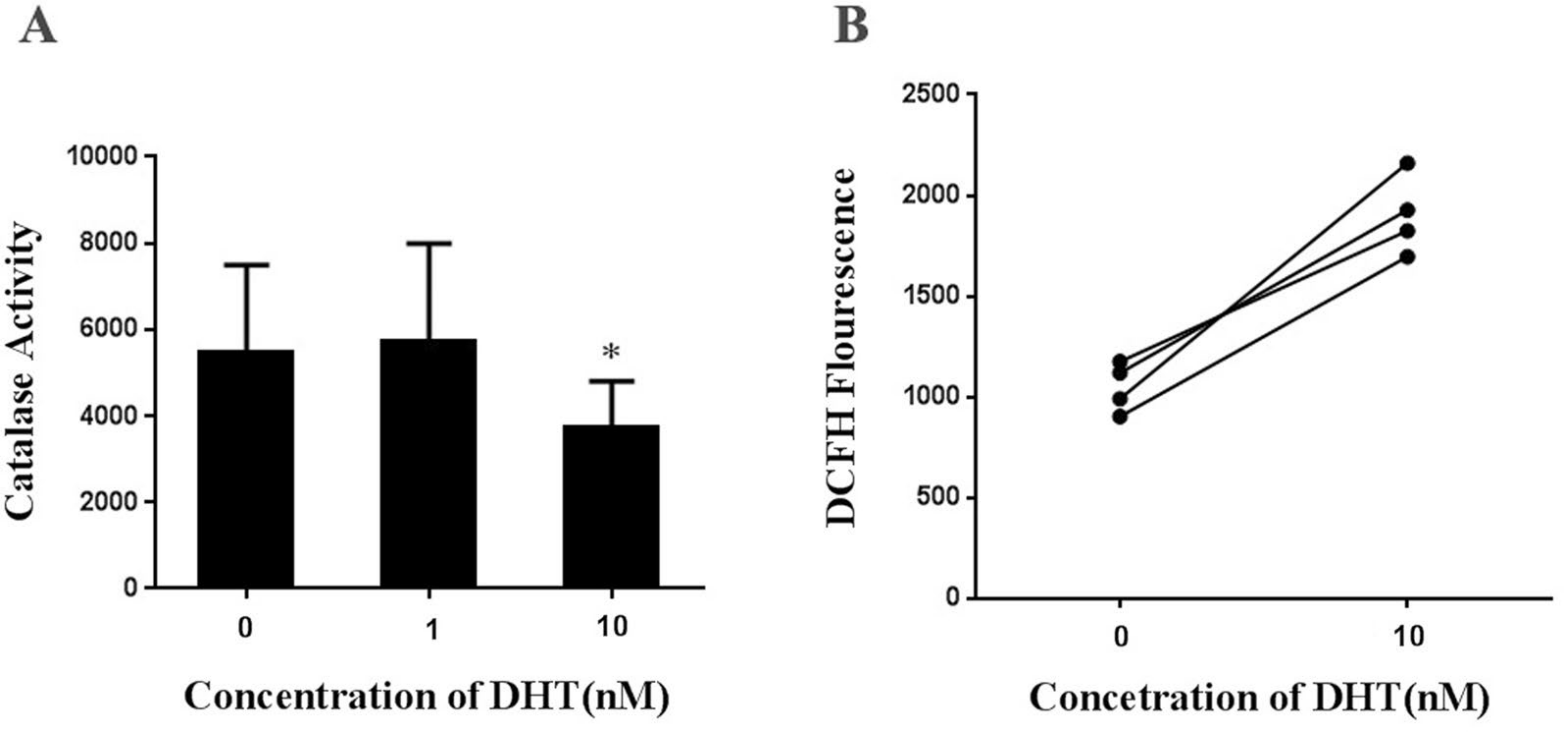
DHT treatment suppressed the catalase activity in LNCaP cells. **A** The catalase activity was decreased in LNCaP cells after DHT treatment for 48 h. **B** The level of ROS increased after DHT treatment for 48 h in LNCaP cells (*p < 0.0001)

To study the effect of DHT on the catalase activity, we tested the catalase activity in LNCaP cells which was treated with DHT (1nM,10nM) for 48h. Compared with the control group, the catalase activity in DHT group (10nM,) was significantly decreased (Fig. 5A). Then we investigated the involvement of ROS by adding DCFH-DA to detect ROS production. We found that the ROS in DHT group was increased by 80% compared with the control group (*p*<0.001) (Fig. 5B).

### ROS scavenger Tiron could inhibit DHT-induced LNCaP cells proliferation

**Fig. 6:**
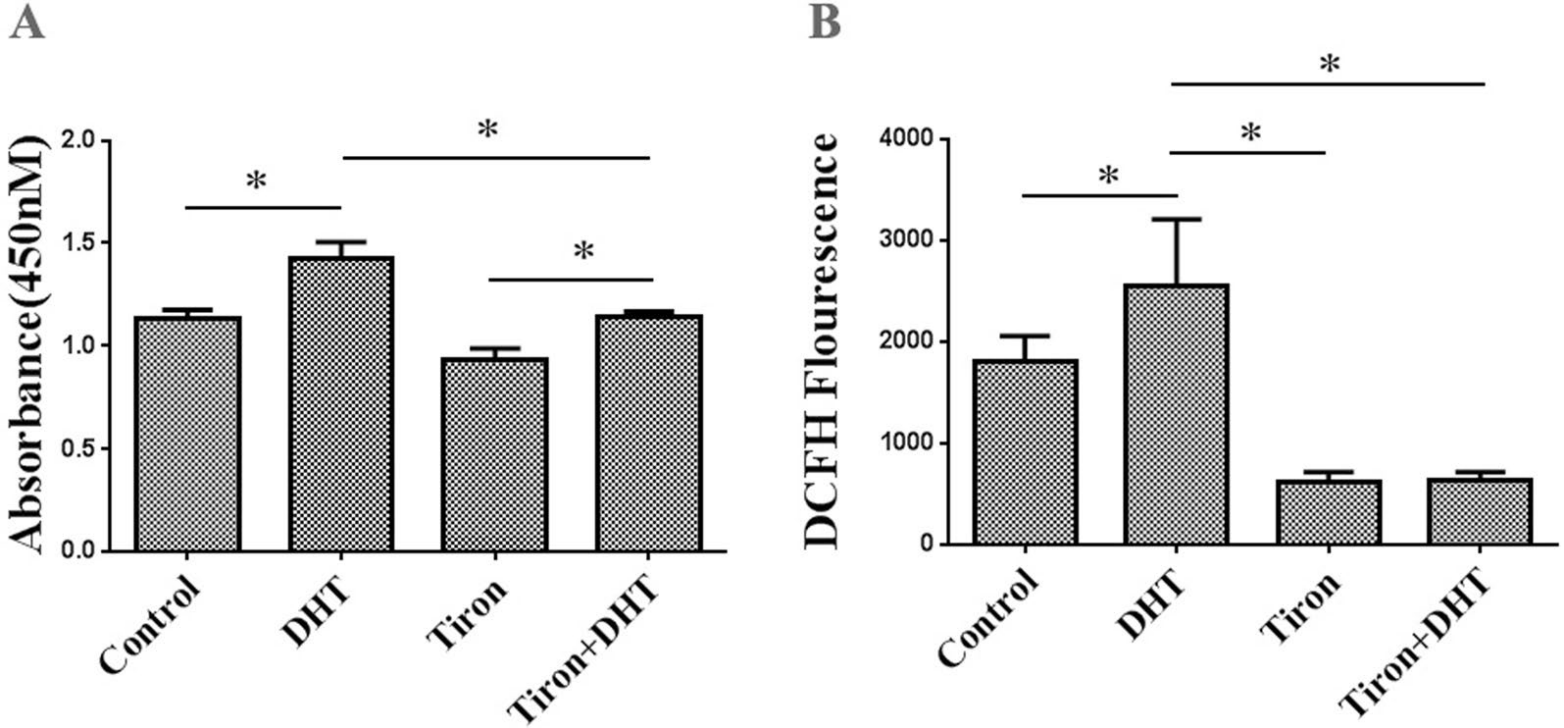
The effect of the superoxide anion scavenger Tiron on the LNCaP cells proliferation. **A** Tiron treatment reduced DHT-induced proliferation by CCK8 assay. **B** Tiron treatment reduced DHT-induced ROS production (*p < 0.05)

ROS production increased in DHT-induced LNCaP cells proliferation. Whether DHT-induced cell proliferation depends on ROS production remains unknown. Herein, we used the ROS scavenger Tiron (1mM) to culture LNCaP cells. We found that Tiron treatment reduced DHT-induced proliferation (Fig. 6A) and ROS production (*p* < 0.05) (Fig. 6B). This data further suggested that DHT promotes cell proliferation by reducing the expression of catalase and increasing ROS production. Besides, ROS producion in the Tiron treatment group also decreased, indicating that Tiron could eliminate intracellular ROS and reduce the prostate cancer cell proliferation.

## Discussion

FOXO3a is a member of the FOXO subfamily of forkhead transcription factors. It can induce most of the cellular processes, such as cell apoptosis, cell proliferation, cell cycle progression, DNA damage, and tumorigenesis. The post-transcriptional inhibition of microRNAs (miRNAs), PTMs and protein–protein interaction can regulate the function of FOXO3a [4].

In this study, we identified a novel mechanism that FOXO3a expression decreasing promotes prostate cancer cell proliferation by activating ROS signaling. Our results also demonstrated that DHT treatment promoted cell proliferation via inhibiting the expression of FOXO3a and arresting the catalase activity, which leads to the ROS production increasing in PCa cell.

Several studies have already reported that FOXO3a was decreased in human prostate cancer and and they found deregulation of FOXO3a could promote prostate cancer progression [5, 9, 15, 16]. Consistent with previous study, our result also demonstrated that DHT or R1881 treatment promoted the proliferation of LNCaP and 22RV1 cells in a dose-dependent manner, and down-regulated the expression of FOXO3a in LNCaP cells. The expression of FOXO3a decreased in an androgen dependent manner in LNCaP cells. These results indicated that FOXO3a played a major role in the PCa cells. Benefiting from the TCGA dataset, we foud that catalase expression was reduced in PCa. Through the GEPIA database, a positive correlation was found between FOXO3a and catatlase. A protein–protein interaction also found between FOXO3a and catalase in the STRING database. Indeed,our study showed that catalase expression was strongly affected by FOXO3a. It consisted with previous study that FOXO3a can bind to the ATAAATA sequence in catalase promoter and then positively regulated catalase expression in rat cell [4, 17, 18]. Consequently, silencing FOXO3a reduce in the catalase mRNA and protein production in rat cardiomyocytes [17, 18]. In addition, our data showed that the mRNA and protein levels of FOXO3a and catalase were significantly decreased in the LNCaP cells after DHT treatment. Ectopic expression and silencing of FOXO3a further suggested that FOXO3a played a regulatory role on the upstream of catalase.

Catalase was encoded by *CAT* gene, and is a key enzyme in the metabolism of H_2_O_2_ and ROS [19]. The expression of catalase is various in human tumors [19–21]. It suggested that different types of cancer cells may showed different sensibility to oxidative stress. We investigated the catalase activity in prostate cancer cell under DHT treatment. Compared to the control group, the catalase activity in the DHT treatment group was significantly reduced. Furthermore, we found that the ROS level in the DHT treatment group was increased compared with control group. Increased ROS production has been observed in various cancers and shows several roles in the cell progress, for example, it could activate pro-tumor signals, and then enhance the cell survival and proliferation [22, 23]. Our observation indicated that the increased ROS by DHT treatment induced the prostate cancer cell proliferation. Some researchers also reported that ROS was involved in the anti-tumor signals by initiating oxidative stress-induced cancer cells death [22–24]. Therefore, it is critical for cancer cells to maintain ROS homeostasis. Intervening ROS homeostasis maybe a promising method for cancer therapy.

Antioxidants showed an vital role in suppressing the activities of ROS [25]. Catalase is one of the endogenous antioxidant [26]. When the endogenous antioxidant system is inactive, reducing the excessive production of ROS depends on exogenous antioxidants, such as Tiron which is one of the ROS scavenger and it could promote cell survival by inhibiting the releasing of cytochrome c, caspase-3 activity [27]. In our study, Tiron reduced the DHT-induced proliferation and ROS production. In summary, our data suggested that DHT promoted cell proliferation by reducing the expression of catalase and then increasing ROS in prostate cancer cell. Tiron could reduce the intracellular ROS and then inhibit the DHT-induced cell proliferation. It was further demonstrated that antioxidant played an important role in cancer chemoprevention by suppressing oxidative stress-induced cell survival.

## Conclusions

Above all, results shown here revealed the mechanisms by which DHT promoted PCa cell proliferation was that FOXO3a suppressed catalase expression and activated ROS signaling.

## Supplementary Information

**Additional file 1.** **Fig. S1.** The effect of R1881 and Enzalutamide (ENZ) on the proliferation of LNCaP cells by CCK8 assay (450 nm).

**Additional file 2.** **Fig. S2.** Enzalutamide (40 μM or 60 μM) could slightly reduce the proliferation of LNCaP and 22RV1 cells by CCK8 assay (450 nm).

## Data Availability

All data analysed during this study are included in this published article. Generated data from each experimental repeat are available from the corresponding author on reasonable request.

## Abbreviations

PCa: Prostate cancer
ROS: Reactive oxygen species
FOXO: Forkhead box O transcription factors
GEPIA: Gene expression profiling interactive analysis
TCGA: The Cancer Genome Atlas
PPI: Protein-protein interaction
DHT: Dihydrotestosterone
DCFH-DA: Dichloro-dihydro-fluorescein diacetate
CRPC: Castration-resistant prostate cancer
qRT-PCR: Quantitative real-time-PCR
PTMs: Post-translational modifications
