# Supplementary figures and images for: FOXO3a-ROS pathway is involved in androgen-induced proliferation of prostate cancer cell
Source: BMC Urol. 2022 Apr 29;22:70. doi: 10.1186/s12894-022-01020-9 (PMC9052560; doi:10.1186/s12894-022-01020-9)

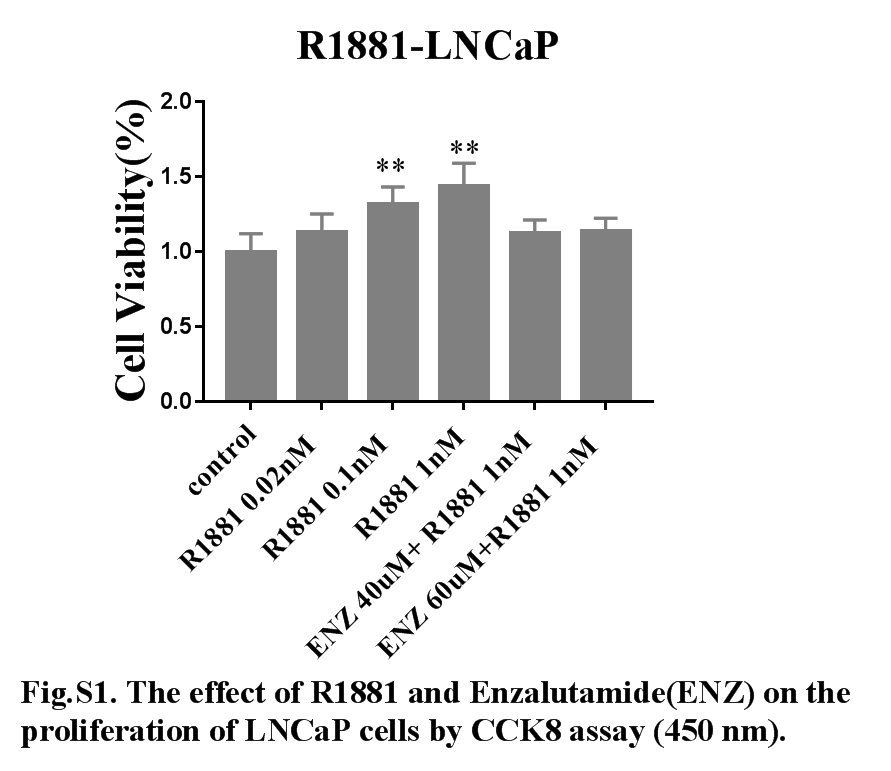

Supplement: Supplementary file 1 — Additional file 1. Fig. S1. The effect of R1881 and Enzalutamide (ENZ) on the proliferation of LNCaP cells by CCK8 assay (450 nm). [file 12894_2022_1020_MOESM1_ESM.tif]

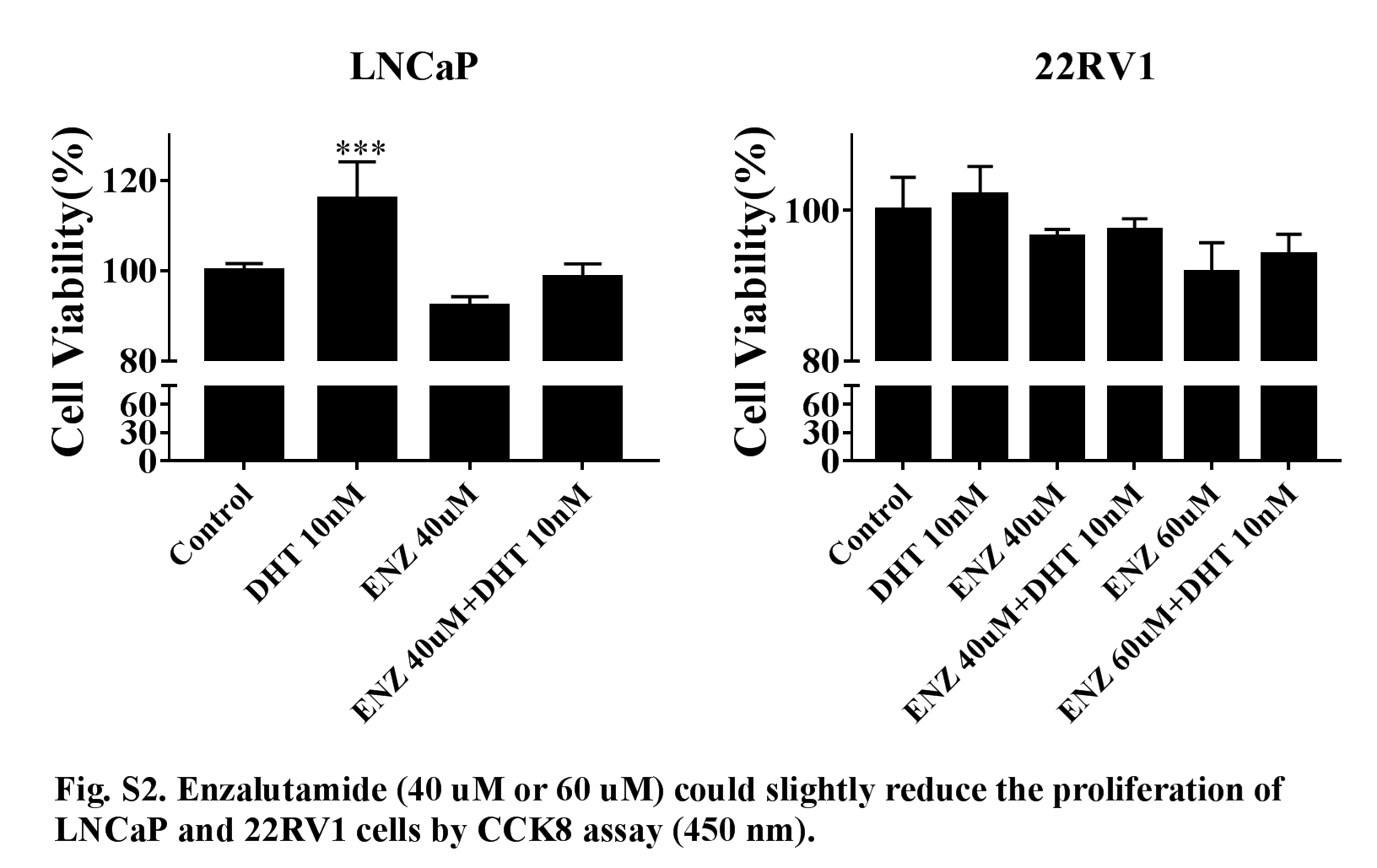

Supplement: Supplementary file 2 — Additional file 2. Fig. S2. Enzalutamide (40 μM or 60 μM) could slightly reduce the proliferation of LNCaP and 22RV1 cells by CCK8 assay (450 nm). [file 12894_2022_1020_MOESM2_ESM.tif]
